# Increased Brain-Derived Neurotrophic Factor in Lumbar Dorsal Root Ganglia Contributes to the Enhanced Exercise Pressor Reflex in Heart Failure

**DOI:** 10.3390/ijms20061480

**Published:** 2019-03-24

**Authors:** Alicia M. Schiller, Juan Hong, Zhiqiu Xia, Han-Jun Wang

**Affiliations:** Department of Anesthesiology, University of Nebraska Medical Center, Omaha, NE 68198-5850, USA; alicia.schiller@unmc.edu (A.M.S.); juan.hong@unmc.edu (J.H.); Zhiqiu.xia@unmc.edu (Z.X.)

**Keywords:** exercise, mechanoreflex, cardiovascular reflexes, neurotrophic factors

## Abstract

An exaggerated exercise pressor reflex (EPR) is associated with excessive sympatho-excitation and exercise intolerance in the chronic heart failure (CHF) state. We hypothesized that brain-derived neurotrophic factor (BDNF) causes the exaggerated EPR via sensitizing muscle mechanosensitive afferents in CHF. Increased BDNF expression was observed in lumbar dorsal root ganglia (DRGs) from CHF rats compared to sham rats. Immunofluorescence data showed a greater increase in the number of BDNF-positive neurons in medium and large-sized DRG subpopulations from CHF rats. Patch clamp data showed that incubation with BDNF for 4–6 h, significantly decreased the current threshold-inducing action potential (AP), threshold potential and the number of APs during current injection in Dil-labeled isolectin B4 (IB4)-negative medium-sized DRG neurons (mainly mechano-sensitive) from sham rats. Compared to sham rats, CHF rats exhibited an increased number of APs during current injection in the same DRG subpopulation, which was significantly attenuated by 4-h incubation with anti-BDNF. Finally, chronic epidural delivery of anti-BDNF attenuated the exaggerated pressor response to either static contraction or passive stretch in CHF rats whereas this intervention had no effect on the pressor response to hindlimb arterial injection of capsaicin. These data suggest that increased BDNF in lumbar DRGs contributes to the exaggerated EPR in CHF.

## 1. Introduction

A hallmark of patients suffering from chronic heart failure (CHF) is sympatho-excitation and exercise intolerance [1,2,3,4]. The degree of exercise intolerance is important to characterize in patients with CHF, since it has implications for morbidity, disability, and prognosis, and is often the reason patients seek medical attention. Surprisingly, evidence suggests that the degree of exercise intolerance is not necessarily related to the degree of cardiac dysfunction [5,6]. Rather, it is generally thought that factors in the peripheral musculature play a critical role in mediating exercise intolerance, including abnormalities in skeletal muscle structure and a neural reflex originating in skeletal muscle, termed the “exercise pressor reflex” (EPR).

During exercise, the cardiovascular system is regulated, in part, by the EPR, a peripheral neural reflex originating in skeletal muscle. The afferent arms of this reflex are composed of metabolically and mechanically sensitive afferent fibers containing predominantly group IV, C-fibers and group III, A-delta fibers, respectively [7,8,9,10]. Evidence from human and animal studies has demonstrated that increases in heart rate, arterial pressure and ventilation in response to activation of this reflex are enhanced in CHF patients and animals [11,12,13,14,15]. Experimental and clinical studies by us [16] and others [11,12,13,14,15] have provided convincing evidence that the enhanced mechanically sensitive afferent input of this reflex (i.e., mechanoreflex) contributes to the exaggerated EPR in CHF while the metaboreflex is blunted. However, further molecular mechanisms underlying the selective sensitization of the mechano-sensitive afferent limb in CHF has not been fully explored.

Brain-derived neurotrophic factor (BDNF), a major growth factor of the central nervous system, is a member of the family of neurotrophins, which also comprises nerve growth factor (NGF) and neurotrophins [17] 3 and 4 [18]. Neurotrophins bind to their cognate tyrosine kinases (Trk) receptor tyrosine kinases TrkA, B or C, to initiate downstream signaling events. Of them, BDNF binds to the low-affinity p75 neurotrophin receptors and the high-affinity tyrosine kinase TrkB receptors [18]. BDNF is normally present in small and medium DRG neurons [19]. Treatment with BDNF can reduce nociceptive thresholds in rats [20] and suppresses the activity of voltage-activated K^+^ (Kv) channels in DRG neurons [21]. Sciatic nerve ligation or axotomy increases the expression level of BDNF in the DRG [22]. However, little is known about whether BDNF is causally involved in the muscle mechanical afferent sensitization in CHF. Therefore, our goal in this study was to explore the role of BDNF in mediating the selective sensitization of the mechanical afferent limb of the EPR in CHF.

## 2. Results

### 2.1. Evaluation of Body Weight, Organ Weight and Baseline Hemodynamics

Echocardiographic parameters at 6 weeks post myocardial infarction (MI) and hemodynamic measurements at 10 weeks post MI in all groups are summarized in Table 1. MI-induced cardiac dilation in CHF + anti-BDNF and CHF + vehicle rats was indicated by increased left ventricle (LV) systolic and diastolic diameters and volumes measured by echocardiography at the 6th week post MI. Furthermore, these 6-week MI rats exhibited reduced ejection fraction and fractional shortening compared with sham rats, indicating decreased cardiac systolic function. Hemodynamic data collected at the time of the terminal experiments (~10 weeks) further demonstrated that there was a significant increase in left ventricle end-diastolic pressure (LVEDP) in CHF rats compared to sham rats. The heart weight and lung weight to body weight ratios were significantly higher in CHF rats compared to sham-operated rats, suggesting cardiac hypertrophy and substantial pulmonary congestion in the CHF state. Moreover, in rats with CHF, a gross examination revealed a dense scar in the anterior ventricular wall. The mean infarct area was 40.2 ± 1.8% of the LV area in CHF + Vehicle and 41.1 ± 1.5% of the LV area in CHF + anti-BDNF (Table 1). No infarcts were identified in sham-operated rats. There was no significant difference in echocardiographic parameters, LVEDP and heart weight and lung weight to-body weight ratios between CHF and CHF + anti-BDNF groups.

### 2.2. Increased BDNF Protein Expression in Lumbar DRGs in CHF Rats

**Figure 1:**
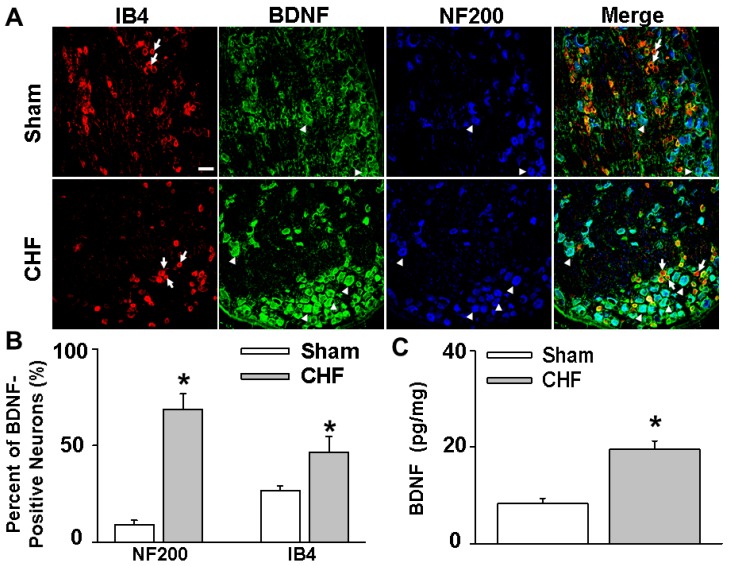
(**A**,**B**) Brain-derived neurotrophic factor (BDNF) expression in lumbar dorsal root ganglia (DRGs) measured by immunofluorescence in sham and CHF rats (*n* = 5/each). Isolectin B4 (IB4, red color), a C-fiber neuron marker; NF200 (blue color), an A-fiber neuronal marker. White bar = 50 μm. White arrows point to the small IB4-positive neurons without BDNF staining (green color). White arrowheads represent double staining of BDNF with NF200, which are medium or large sized soma. (**C**) BDNF expression in lumbar DRGs measured by ELISA assay in sham and CHF rats (mean ± SE. *n* = 6/each). * *p* < 0.05 vs. sham.

An enzyme-linked immunosorbent assay (ELISA) assay was performed to compare the BDNF content in lumbar DRGs between sham and CHF rats. Increased BDNF expression was observed in lumbar DRGs from CHF rats compared to sham rats (Figure 1; 8.3 ± 1.1 vs. 19.5 ± 1.8 pg/mg, *n* = 6, *p* < 0.05). Immunofluorescence data further showed a marked increase in the number of BDNF-positive neurons, especially in medium and large-sized DRG subpopulations (mainly mechanically sensitive NF200 positive neurons) from CHF rats compared to sham rats (8.8 ± 2.5% vs. 68.8 ± 8.1%, *n* = 5/each, *p* < 0.05). In addition, the number of BDNF-positive neurons in small-sized DRG subpopulations (mainly metabolically-sensitive IB4-positive neurons) was also moderately increased in L4-L6 DRGs of CHF rats compared to sham rats (26.8 ± 2.4% vs. 46.4 ± 8.3%, *n* = 5/each, *p* < 0.05).

### 2.3. Effect of BDNF and Anti-BDNF on Muscle Afferent DRG Neuronal Excitability in Sham and CHF Rats

**Figure 2:**
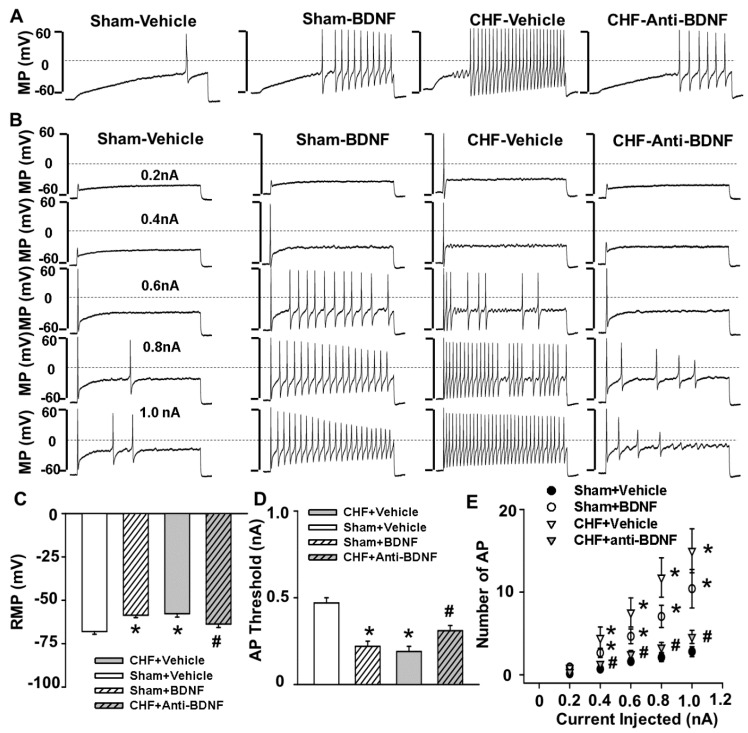
Original tracings (**A**,**B**) and mean data (**C**–**E**) showing the effect of 4–6 h incubation of BDNF and anti-BDNF on action potential properties in response to current injection with either an ramp injection protocol (0–1.0 nA, 1.0 sec) or a step injection protocol (200 pA/step, 0–1 nA, 1.0 sec) in the IB4-negative medium-sized muscle afferent DRG neurons from sham + vehicle, sham + BDNF (100 ng/mL), CHF + vehicle and CHF + anti-BDNF (10 µg/mL) groups. Mean ± SE. *n* = 13–16 neurons from 6–8 rats/each group. * *p* < 0.05 vs. sham. # *p* < 0.05 vs. CHF + Vehicle. AP, action potential. MP, membrane potential; RMP, rest membrane potential.

We used the current-clamp technique to determine the direct effect of BDNF on Dil-labeled IB4-negative medium-sized DRG neurons (i.e., a DRG subpopulation most likely containing muscle mechano-sensitive afferent neurons) in sham rats. Compared to the vehicle group, 4–6 h incubation of BDNF (100 ng/mL) significantly decreased the current threshold-inducing APs or threshold potential in response to a ramp current-stimulation protocol (0–1.0 nA, 1.0 sec) in the DiI-labeled IB4-negative medium-sized muscle afferent DRG neurons in sham rats (Figure 2A,C,D). Furthermore, 4–6 h incubation with BDNF (100 ng/mL) also significantly increased the number of action potentials in response to a step current-stimulation protocol (0–1.0 nA, 200 pA/step over 1.0 sec) in the same DRG subpopulation in sham rats (Figure 2B,E). It should be noted that AP generation in this DRG subpopulation exhibits an interesting “adaptation” phenomenon during a 1-sec static current stimulation (i.e., rapid discharge during the onset of current injection followed by silencing of neuronal firing during the steady state period of current stimulation). This neuronal firing pattern is very similar to the muscle mechanical afferent discharge pattern during static muscle contraction (i.e., a sudden explosive burst of discharge during the onset of static contraction followed by an adaptive decrease during the steady state period of contraction), indicating that the DRG neurons selected most likely originate from a muscle mechanically sensitive afferent DRG subpopulation. However, in BDNF-treated DRG neurons, this “adaption” phenomenon was largely abolished, and their firing discharges were significantly increased and maintained during the total period of static current injection. These data suggest that increased BDNF content may sensitize muscle mechano-sensitive afferent neurons. Compared to sham rats, there was a significant reduction in the current threshold-inducing action potential or threshold potential in response to a ramp current-stimulation protocol (0–1 nA, 1.0 sec) in the Dil-labeled IB4-negative medium-sized DRG population in CHF rats (Figure 2A,C,D). Compared to sham rats, there was also a significant increase in the number of action potentials in response to a step current-stimulation protocol in the same DRG subpopulation in CHF rats (Figure 2B,E). Similar to the BDNF-treated DRG neurons, the neuronal firing “adaption” phenomenon was also abolished in the CHF IB4-negative medium-sized DRG neurons. To the best of our knowledge, these data are the first to suggest that there is increased muscle afferent DRG neuronal excitability in CHF, which is consistent with our previous findings that muscle afferent discharges in response to mechanical stimuli was enhanced in CHF [16]. Finally, we found that 4–6 h incubation with anti-BDNF (10 µg/mL) restored the abnormalities in AP generation in this DRG subpopulation in CHF rats (Figure 2).

### 2.4. Chronic Epidural Infusion of Anti-BDNF Attenuates the Exaggerated EPR in CHF Rats

**Figure 3:**
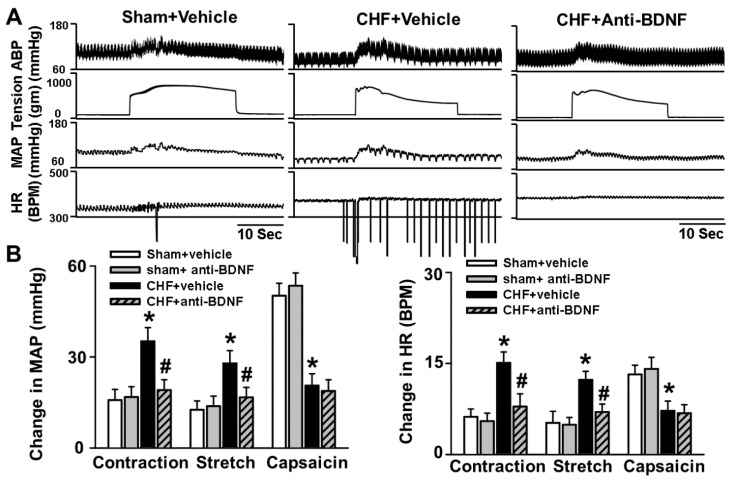
The effect of 7-day epidural infusion of anti-BDNF (1.0 μg/μL/h, 1) to lumbar DRGs on the pressor and tachycardia responses to static contraction, passive stretch or hindlimb arterial infusion capsaicin (1.0 μg/kg, 0.2 mL) in sham and CHF rats. (**A**) The representative figures showed the pressor and tachycardia responses to muscle static contraction in Sham + Vehicle, CHF + Vehicle and CHF + Anti-BDNF rats respectively. (**B**) the summary data showed the pressor and tachycardia responses to static contraction, passive stretch or hindlimb arterial infusion capsaicin in sham and CHF rats with and without anti-BDNF treatment. Mean ± SE. *n* = 6–8/each group. * *p* < 0.05 vs. sham + Vehicle, # *p* < 0.05 vs. CHF + vehicle group.

Previous studies, including our own, have demonstrated the exaggerated pressor response to either static contraction (EPR activation) or passive stretch (mechanoreflex) in CHF rats [11,12,13,14,15], indicating the exaggerated EPR and enhanced mechanoreflex in CHF. Here we show that the exaggerated pressor response to either static contraction (EPR activation) or passive stretch (a pure mechanical stimulus) observed in CHF rats was significantly attenuated by chronic epidural delivery of anti-BDNF to the lumbar DRGs whereas this intervention had no effect on the pressor response to hindlimb arterial injection of capsaicin (metaboreflex activation) (Figure 3), suggesting that increased BDNF in lumbar DRGs contributes to the exaggerated EPR by sensitizing the mechanical afferent limb in CHF rats. Treatment with anti-BDNF in lumbar DRGs of sham rats had no significant effect on the EPR, mechanoreflex or metaboreflex. There was no significant difference in tension-time indexes [23] during either static contraction or passive stretch among groups (Table 2), indicating that the effect of anti-BDNF on the EPR as well as mechanoreflex in CHF rats was not due to the altered muscle tension by anti-BDNF delivery.

## 3. Discussion

The primary new findings of this study are that (1) there is increased BDNF expression in lumbar DRGs of CHF rats compared to sham rats. Data from immunofluorescence experiments further showed a greater increase in the number of BDNF-positive neurons, especially in medium and large-sized DRG subpopulations (mainly mechanically sensitive, marked by NF200), from CHF rats compared to sham rats; (2) 4–6 h incubation with BDNF increased neuronal excitability in Dil-labeled IB4-negative medium-sized muscle afferent DRG neurons (a DRG subpopulation most likely containing mechano-sensitive neurons) in sham rats, whereas anti-BDNF treatment attenuated the enhanced neuronal excitability in the same DRG subpopulation in CHF rats, indicating the possibility that increased BDNF in lumbar DRGs may contribute to increased muscle mechano-sensitive afferent neuronal excitability in CHF; (3) chronic epidural administration of anti-BDNF attenuated the pressor response to either static contraction induced by electrical stimulation of L4/L5 ventral roots (EPR) or passive stretch (a purely mechanical stimulus) in CHF rats but not in sham rats, indicating that BDNF in lumbar DRGs contributes to the genesis of the exaggerated EPR and the mechanoreflex in CHF rats but does not tonically modulate the EPR and mechanoreflex in sham rats; (4) chronic epidural administration of anti-BDNF did not improve the blunted cardiovascular responses to hindlimb arterial injection of capsaicin, indicating that the BDNF signaling pathway in lumbar DRGs is not involved in the mechanisms underlying the blunted metaboreflex in CHF. To the best of our knowledge, these data are the first to highlight the importance of increases in BDNF in peripheral afferent ganglia in mediating the exaggerated EPR in the CHF state.

Trk receptors are a family of three receptor tyrosine kinases (Trk A, B and C), each of which can be activated by one or more of four neurotrophins—nerve growth factor (NGF), BDNF, and neurotrophins 3 and 4 (NT3 and NT4). Neurotrophin signaling through these receptors regulates cell survival, proliferation, the fate of neural precursors, axon and dendrite growth and patterning, and the expression and activity of functionally important proteins, such as ion channels and neurotransmitter receptors [18,24,25]. More recently, attention has been focused on elucidating the role of neurotrophic factors in neuronal network patterning and neuronal sensitivity by facilitating actions such as long-term potentiation [26]. For example, BDNF can function as both a neurotransmitter and neuromodulator and can thus act both acutely and chronically to alter neuronal firing and neuronal sensitivity to synaptic input [27]. Signaling by BDNF and its receptor, TrkB, has long been understood to potentiate synapses through modulating neurotransmitter release, increasing glutamatergic signaling, and increasing excitatory ion channel activity in a large number of neuronal pathways such as in the hippocampus [26]. Furthermore, a recent study from this laboratory also suggested that endogenous BDNF signaling in the nucleus tractus solitarius (NTS) is integral for the maintenance of baroreflex sensitivity in the normal condition [28]. However, there are very few studies to focus on the role of BDNF and other neurotrophins in modulating skeletal muscle afferents in both normal and diseases conditions. In this study, we provide the first evidence that BDNF is also involved in regulation of the muscle afferent DRG neuronal excitability as well as the exaggerated EPR in CHF, suggesting that altered BDNF signaling could also contribute to the neuronal dysfunction in the peripheral sensory nervous system in CHF.

Skeletal muscle is richly endowed with group III and IV afferent nerve fibers which sense the mechanical and metabolic environment. During exercise, muscle mechanoreceptors/group III afferents are immediately stimulated with the increases in muscle tension, whereas stimulation of metaboreceptors/Group IV afferents requires time for activation due to the buildup of interstitial metabolites (e.g., H+, lactate, K+, etc.). Activation of group III and IV afferents elicits changes in autonomic activity which raises arterial pressure, heart rate and sympathetic nerve activation (i.e., the EPR). In animals with CHF and in patients, this reflex is exaggerated and causes extreme activation of the sympathetic nervous system during exercise [11,12,13,14,15]. The exaggerated sympathetic activation by the EPR during exercise restrains muscle blood flow, arteriolar dilation, and capillary recruitment, leading to under perfused areas of working muscle. Furthermore, considering that cardiac contractility and stroke volume have a low capacity to increase during exercise in CHF [29,30], extreme visceral vasoconstriction by the exaggerated EPR may cause fluid shifts from visceral organs to the chest during exercise, which may result in increased risk of acute heart failure and lung edema. The latter can also cause dyspnea and accentuates the symptoms of exercise intolerance. Therefore, exploring the mechanisms underlying the genesis of the exaggerated EPR in CHF has important clinical significance for developing a potential therapy against exercise intolerance and reducing the cardiovascular risk during physical activity in CHF patients. Experimental and clinical studies by us [16] and others have provided convincing evidence that the enhanced mechanically sensitive afferent input of this reflex primarily contributes to the genesis of the exaggerated EPR in CHF. Direct muscle afferent recording experiments by us and others [8,9] has demonstrated that under physiological conditions, muscle mechanical afferents exhibit a “quick adaption” phenomenon during static exercise (i.e., a sudden explosive burst of discharge during the onset of muscle contraction followed by an adaptive decrease during the steady state period of contraction). In CHF animals, this mechanical afferent “adaption” phenomenon is largely abolished and their firing discharge is significantly increased and maintained during the total period of muscle contraction. However, the molecular and cellular mechanisms underlying the selective sensitization of the mechano-sensitive afferent limb in CHF has not been fully elucidated. Recently, several studies from Li and colleagues highlighted the importance of increased NGF in lumbar DRGs to the genesis of the exaggerated EPR by affecting the metaboreflex in peripheral arterial disease [31,32]. It is also of interest to determine if NGF is involved in the sensitivity of the EPR in the CHF state. However, the studies by Garry et al. [33,34] and Xing et al. [35] excluded this possibility because they reported that NGF concentration in lumbar DRGs was decreased in the CHF state. Given that NGF is produced by peripheral tissues such as skin and skeletal muscle and retrogradely transported to DRG soma via axonal flow, this observation suggests a low capacity of peripheral tissues to produce NGF. However, the contribution of other neurotrophins to the genesis of the exaggerated EPR in CHF has not been determined. Unlike NGF, BDNF is mainly produced by DRG neurons and anterogradely transported to the nerve terminal [36,37]. In addition, the high-affinity BDNF receptor (Trk B) is primarily located in medium and large DRG neurons (mainly mechanically sensitive) whereas the high-affinity NGF receptor (Trk A) is primarily located in small DRG neurons (mainly metabolically sensitive) [38,39,40]. Therefore, the possibility exists that alterations of BDNF in DRG may selectively affect the mechanically sensitive DRG subpopulation. Our molecular data demonstrates a greater increase in the number of BDNF-positive neurons, especially in medium- and large-sized DRG subpopulations (mainly mechanically sensitive, marked by NF200), from CHF rats compared to sham rats. Furthermore, our data show that BDNF increased neuronal excitability in Dil-labeled IB4-negative medium-sized muscle afferent DRG neurons in sham rats whereas anti-BDNF treatment attenuated the enhanced neuronal excitability in the same DRG subpopulation in CHF rats, indicating that increased BDNF might contribute to increased muscle mechano-sensitive afferent neuronal excitability in CHF. Finally, chronic epidural delivery of anti-BDNF to lumbar DRGs attenuates the exaggerated EPR and mechanoreflex but not the metaboreflex in CHF rats. These data strongly suggest that BDNF may play a critical role in mediating the exaggerated EPR via sensitizing the mechanical (group III) afferent limb in CHF.

Although the current study did not explore the mechanisms by which BDNF modulates muscle afferent DRG neurons in CHF, several previous studies [20,21,22,41] provide several possibilities. First, Cao et al. [21] reported that (1) 4-h incubation with BDNF caused a large reduction in Kv currents, especially rapidly inactivating ‘transient’ A-type currents (IA), in medium and large DRG neurons from normal rats; (2) the reductions in Kv currents and mRNA levels of IA subunits in lumbar DRGs of diabetic rats were normalized by pre-treatment with an anti-BDNF antibody. Second, our previous study [41] suggested that inhibiting BDNF signaling by pretreating neurons with an anti-BDNF antibody significantly attenuated the Ang II-induced reduction of Kv in a live differentiated catecholaminergic (CATH.a) cell line. It is well known that Kv channels are important for the regulation of the resting membrane potential, the duration and frequency of the AP, and the release of neurotransmitters in neurons [23]. Among the total Kv currents, the I_A_ component is particularly important in the control of the spike onset, the threshold of the AP firing, and the firing frequency [42]. Based on the studies by us and Cao et al. [21,41], it is reasonable to speculate that BDNF increased medium-sized muscle afferent DRG neuronal excitability by suppressing the I_A_ component of the Kv current in CHF. In addition, a study by Obata et al. suggests that increased BDNF resulted in mitogen-activated protein kinase (MAPK) activation in the DRG and caused inflammatory pain [22]. It is well known that MAPK pathways regulate a variety of cellular activities including ion channel function such as Kv channels in DRG neurons [43,44]. Therefore, there may be important effects of BDNF discrete from its neurotrophic properties that impact DRG neuronal excitability and terminal sensitivity by activating MAPK pathways and modulating electrical properties of Kv channels in CHF. However, these hypotheses need to be confirmed in future studies.

## 4. Limitations

We acknowledge that a potential limitation is that our current patch clamp experiments only focused on the role of BDNF on the modulation of mechano-sensitive muscle afferent DRG neurons since the primary goal of this study was to determine the molecular and cellular mechanisms underlying the mechanical afferent sensitization in CHF. However, our data also suggest that BDNF is moderately elevated in IB4-positive C fiber neurons in CHF. Therefore, chronic epidural administration of anti-BDNF antibody could potentially target these DRG neurons in vivo as well. Although we did not observe any significant effect of anti-BDNF on the capsaicin-induced muscle metaboreflex activation in CHF, we cannot rule out the possibility that BDNF may be involved in the modulation of other metaboreceptors on muscle afferents in CHF. For example, our previous study [16] reported that, similar to BDNF, purinergic 2X3 (P2X3) receptor expression in lumbar DRGs was also increased in CHF rats. It remains unclear if increased BDNF contributes to the upregulated P2X3 receptor expression in CHF. Future study will be required to address this issue. In addition, except for BDNF and NGF, the other two primary neurotrophins (i.e., NT-3 and NT-4) may potentially affect the neuronal or sensory activity in both normal and CHF states. These hypotheses remain to be confirmed by additional experiments. Furthermore, since BDNF can bind both TrkB and p75 receptors [18], our current anti-BDNF strategy was not able to differentiate between the TrkB vs. p75 signaling pathways by which BDNF modulates lumbar DRG neurons in CHF. A more specific TrkB/p75 receptor antibody/antagonist strategy will be helpful to answer this question in the future. Finally, in the current study we defined the Dil-labeled IB4-negative medium-sized muscle afferent DRG neurons as muscle mechano-sensitive likely afferent neurons. Although we used IB4 to rule out of the majority of C-fiber DRG neurons from our selected DRG population, a small fraction of IB4-negative peptidergic C fiber neurons may still exist among these neurons. Nevertheless, as shown by Harper et al. [45], the majority of IB4-negative peptidergic C fiber neurons are small-sized (less than 25 µm). Therefore, if there was any peptidergic C fiber neurons selected by us in this medium-sized population, the percentage should be very small. Furthermore, our electrophysiolgical data show that AP generation in our selected DRG subpopulation exhibited a similar “adaptation” phenomenon, which is very close to the muscle mechanical afferent discharge pattern during static muscle. This phenomenon further supports the view that the majority of our selected DRG neurons come from the muscle mechanically sensitive afferent DRG subpopulation.

In summary, our data demonstrate that increased BDNF in lumbar DRGs contributes to the exaggerated EPR by sensitizing the mechanoreflex in CHF rats. Therefore, manipulation of BDNF signaling pathways in DRGs could be considered as a potential new approach to reduce the exaggerated sympatho-exciation and exercise intolerance during exercise in CHF.

## 5. Materials and Methods

Experiments were performed on male Sprague-Dawley rats weighing 420 to 510 g. These experiments were approved (#12-019-04, 5 February 2015) by the Institutional Animal Care and Use Committee of the University of Nebraska Medical Center and were carried out under the guidelines of the National Institute of Health *Guide for the Care and Use of Laboratory Animals*.

### 5.1. Model of CHF

CHF was induced by a procedure used in previous studies conducted by our laboratory [46]. Myocardial infarction was produced by ligating the left coronary artery. A sham group was created in a similar fashion without coronary artery ligation. In brief, rats were anesthetized with 3% isoflurane and ventilated at 60 breaths/min. A left-sided thoracotomy was created level with the fifth intercostal space. The pericardium was opened, the heart was exteriorized, and the left anterior descending coronary artery ligated near its origin. All sham rats survived, but there was a ~30% mortality in the rats that were subject to coronary artery ligation.

In this study, cardiac function in all experimental animals was measured by echocardiography (VEVO 770, Visual Sonics, Inc., Toronto, ON, Canada) as previously described [47,48]. Echocardiographic assessment of cardiac function involved measurement of left ventricular diameters, volumes, ejection fraction and fractional shortening using standard formulas [47,48]. In addition, at the beginning of acute experiments, a Millar catheter (SPR 524; size, 3.5-Fr; Millar Instruments, Houston, TX, USA) was advanced through the right carotid artery into the left ventricle (LV) to determine LV end-diastolic pressure (LVEDP) and LV systolic pressure. The transducer was then pulled back into the aorta and left in place to record blood pressure. At the completion of the experiment the rats were euthanized with an overdose of pentobarbital sodium. The hearts and lungs were removed, and the ratio of the infarct area to whole LV minus septum was measured.

### 5.2. Acute Surgical Preparation

Rats were anesthetized with 3–5% isoflurane (Halocarbon Labs., River Edge, NY, USA), and the jugular vein and trachea were cannulated. Next, the rats were ventilated with 2–3% isoflurane and O_2_. Body temperature was maintained at 37–38 °C by a heating pad. Finally, the right carotid artery was cannulated and AP, mean arterial pressure (MAP) and heart rate (HR) were continuously monitored and recorded.

### 5.3. Decerebration

The decerebration procedure was carried out as described by us and others [46,49,50]. In brief, immediately following the surgical preparation above, the rats were placed in a stereotaxic apparatus (Stoelting Co., Chicago, IL, USA) and custom spinal frame. Dexamethasone (0.2 mg IV) was administered to reduce inflammation and cerebral edema resulting from the decerebration procedure. The non-cannulated carotid artery was ligated and then a portion of bone superior to the central sagittal sinus removed. Next, the cerebral cortex was aspirated to visualize the superior and inferior colliculi. Finally, the brain was perpendicularly sectioned at the pre-collicular level and the transected forebrain aspirated. After the decerebration, the lungs were ventilated with a mixture of room air and oxygen instead of isoflurane. The rats were allowed a minimum of 1.25 h post-decerebration to recover before data collection began.

### 5.4. Activation of the EPR, Mechanoreflex and Metaboreflex

Static hindlimb contraction via stimulation of the ventral roots was used to activate the mechanically and metabolically sensitive skeletal muscle afferent fibers. After performing a laminectomy to expose the lower lumbar portion of the spinal cord (L2–L6), all muscles of the hindlimb were denervated except for the triceps surae. Next, electrically induced static muscle contractions of the triceps surae were produced by simulating the peripheral end of L4/L5 ventral roots for 30 s. Hindlimb stimulation was provided by constant-current at three times motor threshold (defined as the minimum current required to produce a muscle twitch) with a pulse duration of 0.1 ms at 40 Hz.

In decerebrate rats, selective activation of mechanically sensitive intramuscular afferents (group III) was performed as described in previous studies using passive stretch, a pure mechanical stimulus [14,50]. Thus, in animals where ventral root stimulation was performed, we preferentially activated the mechanoreflex by passively stretching the triceps surae muscles using a calibrated rack and pinion system (Harvard Apparatus, Inc., Holliston, MA, USA). Every effort was made to match the peak tension developed in response to electrical stimulation during the passive stretch experiments.

It has been reported that the capsaicin-sensitive receptor (transient receptor potential vanilloid 1; TRPv1) is localized primarily to metabolically sensitive afferent fibers (group IV) in skeletal muscle. As such, the exogenous chemical, capsaicin can be used to preferentially activate these fibers. Therefore, in animals in which ventral root stimulation and passive stretch were performed, hindlimb arterial bolus injections of capsaicin (1.0 µg/kg, 0.2 mL) was used to preferentially activate the metaboreflex [13]. We accomplished this by placing a catheter in the right iliac artery and advancing the tip to the abdominal aortic bifurcation, in order to ensure delivery of capsaicin to the left hindlimb through the left iliac artery. In order to maximally trap capsaicin in the left hindlimb, the common iliac vein was reversibly ligated by a vascular occluder (Harvard, Cambridge, MA, USA). The vein was occluded for 2 min starting immediately before the injection to trap the injectate in the limb. All rats underwent ventral root stimulation, passive stretch, and injection of capsaicin, with the order between these maneuvers randomized and performed at 15-min intervals.

### 5.5. Chronic Epidural Infusion of Anti-BDNF

Rats were anesthetized with isoflurane (2% in O2). They were placed in the prone position and a small midline incision was made in the region of the T13 thoracic to 3rd lumbar vertebrae. Following dissection of the superficial muscles, a small hole was made in the intervertebral space between T13–L1. A polyethylene catheter (PE-30) connected to a Hamilton microinjection syringe (Hamilton company, Reno, NV, USA) was inserted into the lumbar epidural space and gently advanced about 3.5–4.0 cm caudally so that the catheter tip reached the region of the L5 vertebrae. One bolus injection of anti-BDNF (10 µg/mL, 10 µL/per ganglion, Alomone labs, Jerusalem, Israel) was performed around the left L5 DRG and then the catheter was pulled back about 0.8 cm to reach L4 DRG with another injection. After both injections, the PE-30 catheter was secured and connected with a subcutaneous Alzet (Cupertino, CA, USA) osmotic pump (Model 1007D, 0.5 μL/h 7 day capacity) for continuous infusion. A silicone gel (Kwik-Sil; World Precision Instruments, Sarasota, FL, USA) was used to seal the hole between the T13-L1 vertebrae. The skin overlying the muscle was closed using 3-0 polypropylene suture. Simple interrupted sutures were used to close the skin. Betadine was applied to the wound and the rats were allowed to recover from the anesthesia. For post-procedure pain management, buprenorphine (0.05 mg/kg) was subcutaneously injected immediately after surgery and twice daily for 2 days. Terminal experiments were carried out 1 week post infusion.

### 5.6. In Vitro Electrophysiological Recording

#### 5.6.1. DiI Injection into Hindlimb Muscle

We injected the fluorescent retrograde tracer 1,1-dioctadecyl-3,3,3,3-tetramethylindocarbocyaninepercholate (DiI; 20 mg·mL^−1^; Molecular Probes, Eugene, OR, USA) into triceps surae muscle in order to label muscle afferent DRG neurons. Male Sprague–Dawley rats were anaesthetized by inhalation of an isoflurane–oxygen mixture (3–5% isoflurane in oxygen). The skin was incised and the underlying muscle exposed. A total of 5 μL of DiI was injected into the triceps surae muscles, and the needle left in place for 5 to 10 min to minimize tracer leakage. The injection was performed three times (rostral, middle and caudal). The skin was then sutured, and rats returned to their cages for 4–5 days, to allow transportation of the tracer to the DRG neurons.

#### 5.6.2. Isolation of DRG Neurons

After anesthesia with pentobarbital sodium (80 mg/kg, i.p.), the rats were decapitated. DRGs at L4–L6 were removed on the side ipsilateral to the muscle injected with DiI, and placed in ice-cold Ringer’s solution (mM): NaCl, 137; NaHCO3, 25; KCl, 3; NaH2PO4, 1.25; CaCl2, 1.2; MgSO4, 1.2; glucose, 10. The DRGs were dissected free and incubated for 30 min at 37 °C in an enzymatic Ringer’s solution containing 0.1% collagenase/0.1% trypsin (Sigma-Aldrich, St Louis, MO, USA). Next, the tissues were mechanically triturated and immediately transferred to Ringer’s solution containing 0.2% collagenase and 0.5% bovine serum albumin (BSA) and incubated for 30 min at 37 °C. The dispersed DRG cells were then washed with Dulbeccos’s modified Eagle’s medium (DMEM; Gibco, Carlsbad, CA, USA) containing 6% BSA.

The isolated cells were resuspended in culture medium and plated onto culture wells. The culture medium consisted of a 50/50 mixture of Delbecco’s modified Eagle’s medium (DMEM) and Ham’s F12 medium supplemented with antibiotics and 10% fetal bovine serum. The DRG cells were cultured at 37 °C in a humidified atmosphere of 95% air 5% CO2 for 4 to 6 h before the patch clamp experiments.

#### 5.6.3. Electrophysiological Recordings

**Figure 4:**
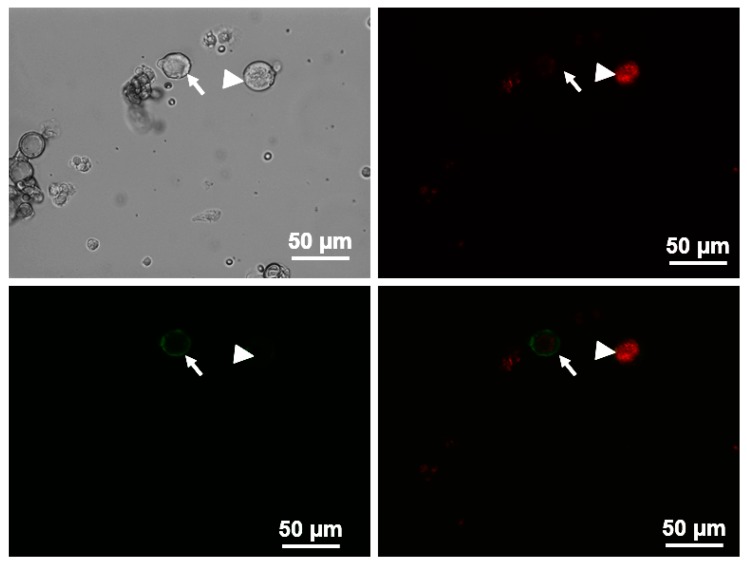
Representative images showing how to identify the medium-sized IB4-negative muscle afferents neurons in dorsal root ganglion (DRG) during patch-clamp experiments. In the top panel, DiI-labeled muscle afferents DRG neurons with red color in fluorescent light (right) and cells in the same field under regular light (left). In the bottom panel, Isolectin B4 (IB4, green color) was used to further separate muscle afferents DRG neurons into two groups: IB4-positive neurons (pointed by white arrow) and IB4-negative neurons (pointed by white arrow head). Digitally merged images from top right panel and bottom left panel are displayed in bottom right panel.

Previous studies have reported that the DRG soma with Aδ (group III) afferents were primarily restricted to medium sized cells (25–35 μm) within the ganglia [45]. Therefore, action potentials (AP) were recorded using the whole-cell patch-clamp technique using Axopatch 200B patch-clamp amplifier (Axon Instruments, Burlingame, CA, USA) only in DiI-labeled medium-sized DRG neurons. To further separate the potential small portion of overlapping C-fiber neurons from these medium-sized DRG population, we used Griffonia simplicifolia isolectin B4 (IB4, Alexa Fluor^®^ 488 conjugate, Invitrogen, Carlsbad, CA, USA), a C fiber marker, to divide the medium-sized DRG neurons into two groups: IB4-positive and IB4-negative groups. Only DiI-labeledIB4-negative medium-size DRG neurons were considered as potential Aδ (group III afferents) neurons. Briefly, DRG neurons were incubated with IB4 for 20 min before recording. The IB4-stained neurons were easily recognized under epifluorescence illumination (Figure 4). In the current-clamp experiments, APs were elicited by a ramp current injection of 0–1 nA and the current threshold-induced APs or threshold potential was measured at the beginning of the first AP. The frequency of APs was measured in a 1-s current clamp (200-pA step decrements from 0 to 1.0 nA for 1 s). Input resistance (R_in_) was determined from the linear fit of the neuronal voltage response to hyperpolarizing current injections (50-pA step decrements from −1.0 to 0 nA for 1 s). There were no significant differences in R_in_ of IB4-negative medium-sized DRG neurons among each group (Sham + Vehicle, 40.8 ± 1.6 MΩ; Sham + BDNF, 48.4 ± 3.5 MΩ; CHF + vehicle, 50.2 ± 2.5 MΩ; CHF + anti-BDNF, 51.5 ± 2.5 MΩ). The patch pipette solution was composed of (in mM) 105 K-aspartate, 20 KCl, 1 CaCl2, 5 MgATP, 10 HEPES, 10 EGTA and 25 glucose (pH 7.2; 320 mosM). The bath solution was composed of (in mM) 140 NaCl, 5.4 KCl, 0.5 MgCl2, 2.5 CaCl2, 5.5 HEPES, 11 glucose and 10 sucrose (pH 7.4; 330 mosM). The P-clamp 10.2 program (Axon Instruments, San Jose, CA, USA) was used for data acquisition and analysis. All experiments were done at room temperature (22–24 °C). BDNF (100 ng/mL) and anti-BDNF (10 µg/mL, Alomone labs, Jerusalem, Israel) in the culture medium were incubated for 4 to 6 h to investigate whether this treatment could alter muscle afferent neuronal excitability in sham and CHF rats.

### 5.7. BDNF Assay

Rats (*n* = 6/each group) were anesthetized with pentobarbital sodium (40 mg/kg, i.p.). L4–L6 DRGs were rapidly excised, weighed and snap frozen in liquid nitrogen prior to storage at −70 °C. Within two weeks of freezing, all collected DRGs were homogenized on the same day in ice cold homogenization buffer consisting of 100 mM Tris/HCl, pH 7, containing 2% bovine serum albumin (BSA), 1M NaCl, 4 mM EDTA.Na2, 2% Triton X-100, 0.1% sodium azide and the protease inhibitors (Sigma) 5 µg/mL aprotinin, 0.5 µg/mL antipain, 157 µg/mL benzamidine, 0.1 µg/mL pepstatin A and 17 µg/mL phenylmethyl-sulphonyl fluoride. The homogenates were centrifuged at 14,000× *g* for 30 min. The resulting supernatants were used for the BDNF assay. ChemiKine BDNF kit (CYT306, Millipore, Burlington, MA, USA) was used to measure the amount of BDNF in rat DRG homogenates following their manual instruction. Each sample was tested in duplicate.

### 5.8. Immunohistochemistry

Rats (*n* = 5/group) were anesthetized with pentobarbital sodium (40 mg/kg, i.p.). After cardiac function was first determined, the rats were perfused with 4% paraformaldehyde (PFA). The L4–L6 DRGs were immediately dissected and post-fixed in 4% PFA overnight. Sections (14 μm) were cut using a cryostat after dehydration with 30% sucrose. After being treated with 10% donkey serum (Jackson ImmumoResearch, West Grove, PA, USA) for 60 min, sections were incubated with rabbit anti-BDNF antibody (1:200 dilution, Alomone labs, Jerusalem, Israel) and mouse anti-NF200 antibody (an A-fiber neuron marker [51], 1:200, Abcam, Cambridge, MA, USA) overnight at 4 °C. Sections were then incubated with Alexa 488-conjugated goat anti-rabbit IgG (1:200, Invitrogen, Carlsbad, CA, USA), pacific blue-conjugated goat anti-mouse IgG (1:200, Invitrogen) and Alexa FluorR568 conjugated isolectin-B4 (a C-fiber neuron marker [17], 1:200, Life Tech Inc., Stafford, TX, USA) for 60 min at room temperature. After being washed with PBS, sections were coverslipped with Aqua-Mount Mounting Medium (VWR Corp., Radnor, PA, USA) and were examined with a laser confocal microscope (Model TSC STED; Leica, Buffalo Grove, IL, USA). Every seventh section (10 sections per rat) was used for consecutive cell quantification analyses using Image J software (https://imagej.nih.gov/ij/download.html). The percent of neurons positive for BDNF for A-fiber or C-fiber neurons was calculated then used for statistical analysis. No staining was seen when a negative control was performed with PBS instead of the primary antibody (data not shown).

### 5.9. Data Acquisition and Statistical Analysis

PowerLab (Model 16S) and LabChart software (version 7.0) (ADInstruments, Colorado Springs, CO, USA) was used to obtain MAP, HR and muscle tension. The peak response from each was determined by the difference of the greatest change from the baseline value. Baseline values were calculated by averaging 30 s of data before the muscle contraction. Tension-time index was calculated by integration of the area between the tension trace and the baseline level expressed in kg × s. Peak developed tension was calculated as the difference between the resting tension from the peak tension (g). All values are expressed as mean ± standard error of the mean (SEM). Differences between groups were determined by a two-way ANOVA followed by the Tukey post hoc test. *p* < 0.05 was considered statistically significant.

## Figures and Tables

**Table 1:** Hemodynamic and morphological data in sham and chronic heart failure (CHF) rats.

Parameters	Sham + Vehicle (*n* = 6)	Sham + Anti-BDNF (*n* = 6)	CHF + Vehicle (*n* = 8)	CHF + Anti-BDNF (*n* = 7)
Body weight, g	447 ± 11	432 ± 12	462 ± 10	451 ± 13
Heart weight, mg	1382 ± 25	1334 ± 27	2190 ± 62 *	2213 ± 58 *
HW/BW, mg/g	3.1 ± 0.1	3.1 ± 0.1	4.7 ± 0.2 *	4.9 ± 0.2 *
WLW/BW, mg/g	4.2 ± 0.3	4.3 ± 0.3	8.7 ± 0.5 *	9.1 ± 0.6 *
MAP, mmHg	106.2 ± 3.3	109.3 ± 2.6	90.2 ± 3.6 *	92.6 ± 3.1 *
HR, bpm	362.2 ± 10.5	348.8 ± 15.9	354.3 ± 11.2	369.3 ± 8.3
LVEDP, mmHg	3.9 ± 0.8	3.2 ± 1.0	17.5 ± 1.5 *	18.8 ± 1.0 *
EF, %	72.5 ± 1.3	74.2 ± 1.4	43.5 ± 1.4 *	42.3 ± 1.4 *
FS, %	42.7 ± 0.7	43.4 ± 0.8	21.7 ± 0.8 *	21.0 ± 0.8 *
Infarct size, %	0	0	40.2 ± 1.8 *	41.1 ± 1.5 *

Values are mean ± SE. BW, body weight; HR, heart rate; HW, heart weight; MAP, mean arterial pressure; WLW, wet lung weight; LVEDP, left ventricle end-diastolic pressure; EF, ejection fraction; FS, fractional shortening. * *p* < 0.05 vs. sham.

**Table 2:** Tension-time indexes for either static contraction or passive stretch in Sham + Vehicle, Sham + Anti-BDNF, CHF + Vehicle and CHF + Anti-BDNF rats.

Group	TTI (kg × s)	
	Contraction	Passive Stretch
Sham + Vehicle	14.7 ± 1.7 (6)	15.8 ± 1.3 (6)
Sham + Anti-BDNF	15.4 ± 1.6 (6)	15.3 ± 1.3 (6)
CHF + Vehicle	14.1 ± 1.5 (8)	15.1 ± 1.1(8)
CHF + Anti-BDNF	14.3 ± 1.4 (7)	16.1 ± 1.4 (7)

Values are mean ± SE, “*n*” in the bracket represents the number of tested fibers. TTI, tension-time index. There were no significant differences (*p* > 0.05) in TTI in all four groups.
